# DTI-BERT: Identifying Drug-Target Interactions in Cellular Networking Based on BERT and Deep Learning Method

**DOI:** 10.3389/fgene.2022.859188

**Published:** 2022-06-08

**Authors:** Jie Zheng, Xuan Xiao, Wang-Ren Qiu

**Affiliations:** Computer Department, Jing-De-Zhen Ceramic Institute, Jing-De-Zhen, China

**Keywords:** drug-target interactions, bidirectional encoder representations from transformers, BRL block, convolutional neural network, computational methods

## Abstract

Drug–target interactions (DTIs) are regarded as an essential part of genomic drug discovery, and computational prediction of DTIs can accelerate to find the lead drug for the target, which can make up for the lack of time-consuming and expensive wet-lab techniques. Currently, many computational methods predict DTIs based on sequential composition or physicochemical properties of drug and target, but further efforts are needed to improve them. In this article, we proposed a new sequence-based method for accurately identifying DTIs. For target protein, we explore using pre-trained Bidirectional Encoder Representations from Transformers (BERT) to extract sequence features, which can provide unique and valuable pattern information. For drug molecules, Discrete Wavelet Transform (DWT) is employed to generate information from drug molecular fingerprints. Then we concatenate the feature vectors of the DTIs, and input them into a feature extraction module consisting of a batch-norm layer, rectified linear activation layer and linear layer, called BRL block and a Convolutional Neural Networks module to extract DTIs features further. Subsequently, a BRL block is used as the prediction engine. After optimizing the model based on contrastive loss and cross-entropy loss, it gave prediction accuracies of the target families of G Protein-coupled receptors, ion channels, enzymes, and nuclear receptors up to 90.1, 94.7, 94.9, and 89%, which indicated that the proposed method can outperform the existing predictors. To make it as convenient as possible for researchers, the web server for the new predictor is freely accessible at: https://bioinfo.jcu.edu.cn/dtibert or http://121.36.221.79/dtibert/. The proposed method may also be a potential option for other DITs.

## 1 Introduction

In the process of drug development, there are many important drug-related interaction directions, including drug-protein, drug-miRNA, drug-disease, drug-drug, etc. Small molecule therapeutic drugs typically exert their effects through binding to one or a few protein targets (Dubach et al., 2014; Lim et al., 2021), therefore identifying drug-protein interaction is an important part of genomic drug discovery (Yamanishi et al., 2014). Besides, several studies have indicated that although ncRNAs lack the potential to encode proteins, they play important roles in cellular functions, and their deregulation heavily contributes to various pathological conditions. Among them, miRNAs are promising therapeutic targets for complex diseases (Wang and Chen, 2019; Yin et al., 2019; Zhou et al., 2020), it thus becomes important to understand the relationship between ncRNAs and drug targets, what’s more, several databases and studies are actively promoting development (Chen et al., 2017). Drug-disease and drug-drug interaction play a crucial role in drug relocation, often serving as important information other than drug-target protein pairing and mainly based on a processing framework called a heterogeneous network. Qu et al. developed a novel computational model of HeteSim-based inference for SM-miRNA Association prediction by implementing a path-based measurement method of HeteSim on a heterogeneous network combined with known miRNA-SM associations, integrated miRNA similarity, and integrated SM similarity (Qu et al., 2019). Jin et al. combine drug features from multiple drug-related networks, and disease features from biomedical corpora with the known drug-disease association’s network to predict the correlation scores between drug and disease (Qu et al., 2019). Drug-protein interactions play a key role in the field of biochemistry due to their scientific significance in drug discovery. This paper focuses on the identification of drug-protein interactions.

Drugs modulate the biological functions of proteins by interacting with target proteins, such as ion channels, nuclear receptors, enzymes, and G Protein-coupled receptors (GPCRs). For an in-depth understanding of the functions of drugs, the knowledge of their target protein is indispensable. Despite the substantial effort, only a few DTIs have been identified so far, since the experimental determination of drug-target interactions remains some defects, such as expensive, time-consuming, low accuracy, and so on (Haggarty et al., 2003). It is highly demanded to develop powerful computational tools, which are capable of detecting potential DTIs. Computational prediction of DTIs has emerged for 20 years as a research hotspot, which is not only for better understanding of the molecular mechanism of drug side effects but also for inventing new genomic drugs and identifying new targets for existing drugs (Wang et al., 2010; Kotlyar et al., 2012).

Knowledge of genomic space and chemical space is indispensable for identifying DITs. With the coming of the post-genome era and the emergence of molecular medicine, transcriptome, and chemical compound, the rapidly increasing knowledge in the field of genomic space and chemical space enables researchers to study drug-target interaction problems (Dobson, 2004) on the basis of high-throughput experimental projects. Several different professional databases have been established, such as Drug Bank, which is consist of two parts information involving drug data and drug target information (Wishart et al., 2018); Therapeutic Target Database (TTD) provides comprehensive information about the drug resistance mutations, gene expressions, and target combinations data (Qin et al., 2014); BindingDB a public database of protein-ligand binding affinities (Liu et al., 2007); Kyoto Encyclopedia of Genes and Genomes (KEGG) including experimental knowledge on protein and their drug target, etc. These resources provide important materials for researchers to predict drug-target interactions based on computational methods, it is time to develop more integrative approaches capable of taking genomic space, chemical space, and the available known drug-target network information into account simultaneously for the issue.

The development of identifying DTIs followed four main directions for research. Firstly, the most direct method is to use the docking simulation (Pujadas et al., 2008; Morris et al., 2009), which is a process of scoring favorable intermolecular interactions, the three-dimensional (3D) structures of proteins and chemical compounds are indispensable. With the development of techniques (e.g., X-ray crystallography, nuclear magnetic resonance), the rate of 3D protein structure determination is increasing every year, however, it is still not able to keep up with the exponential growth of sequence discovery, such as the PDB database only covers a small fraction of the ion channels and GPCRs, both are considered as the most pharmaceutically useful drug targets. Some programs and webservers provide the prediction of the protein structure, in practice, structure prediction is still relatively immature, and interaction prediction may be affected by the inaccurate structure. Secondly, based on the fact that similar molecules usually bind to similar proteins, it is most straightforward to apply the ligand-based approach (Keiser et al., 2007), for example, conducting Quantitative Structure-Activity Relationship (QSAR) studies that a new ligand can be categorized and compared to known proteins ligands. However, ligand-based approaches often present unreliable results due to available binding ligands of targets’ insufficient number, and difficult to scientifically set thresholds to divide positive and negative samples (Butina et al., 2002). Thirdly, literature text mining could be used to extract DTIs from the related articles (Zhu et al., 2005), but this approach could not be used for new drugs and proteins. Fourthly, to overcome the drawbacks of the above-mentioned traditional approaches, chemogenomic approaches are universally studied directions. Chemogenomic approaches integrate information of chemical space, genomic space, and known drug-target interactions, which provide an architecture for deep learning approaches.

Chemogenomic approaches can be classified into three categories: graph-based approaches (Chen et al., 2012), network-based approaches (Alaimo et al., 2013), and learning-based approaches (Mousavian and Masoudi-Nejad, 2014). In the graph-based approach, drugs and targets are represented with graphs, in which nodes for chemical elements or amino acids and adjacency matrices for edges between nodes, adjacency matrices including atom/bond or residue/bond information (Lim et al., 2021). Drug and target graphs can be fed into Graph Neural Network (GNN); after a set of training iterations, information learned by Graph Convolutional Network (GCN) can be converted into vectors for DTIs prediction. Torng and Altman proposed a graph-convolutional framework to determine the interaction patterns (Torng and Altman, 2019). Karlov et al. used the message passing neural network to overcome the limitation of graph convolutional network by considering both nodes and edges (Karlov et al., 2020). Furthermore, the self-attention mechanism in Neural Networks is often coupled with Graph convolutional network to predict DTIs better. But some research showed that there are difficulties in predicting the local non-covalent interactions between drugs and proteins (Li et al., 2020). Network-based approaches utilized the DTI network of identified edges between drugs and targets to identify new DTIs. Indeed, by constructing a heterogeneous network that includes information on drugs, proteins, diseases, and side-effects, the DTINet method can improve the accuracy of DTIs prediction (Luo et al., 2017), but the learning model only takes relatively simple log-bilinear functions, obtaining features may not be the inherent representations of drugs or targets for the final DTI prediction task (Wan et al., 2019). Supervised learning-based approaches are classified into similarity-based approaches and feature-based approaches (Chen et al., 2018). Similarity-based approaches generate the similarity matrixes for drugs and targets respectively, via various similarity measurement strategies such as chemical-based similarity (Haggarty et al., 2003), pharmacological-based similarity (Kim et al., 2013), therapeutic-based similarity, and drug-drug interaction similarity for drugs, and sequence-based similarity (Yamanishi et al., 2008), functional-bases similarity, protein-protein interaction similarity for targets. These similarity matrices have been used in bipartite local models (Mei et al., 2013), matrix factorization models (Ezzat et al., 2016), and the nearest neighbor methods (Zhang et al., 2016) to predict DTIs. The feature-based approaches extract more useful information from protein sequences and drug chemical structure, via the adequate support offered by the rapid development of algorithms.

Predicting DTIs with machine learning algorithms has recently become the focus of research. There are 1-D, 2-D, and 3-D representations of drugs (Rognan, 2007). Simplified Molecular Input Line Entry System (SMILES) string is a typical 1-D representation of the drug (Öztürk et al., 2016) that are commonly used descriptors (Kombo et al., 2013; Sawada et al., 2014). For targets, the sequences of protein are encoded by the physicochemical properties of amino acids, sequential evolution information formulation and general form of pseudo amino acid composition (Li et al., 2020). Lastly, machine learning algorithms are applied for decision-making. Recently, Wang et al. used a novel bag-of-words model and discrete Fourier transform to extract target sequence feature and molecular fingerprint pattern information, respectively, and then use a distance-weighted K-nearest-neighbor algorithm as a predictor (Wang et al., 2020). This paper motivates our work, that instead of using amino acid physic-chemical properties to encode words and perform clustering, we can vectorization drugs and protein by using advanced methods such as word2vec and ProtBert(Elnaggar et al., 2021), which could map every word (amino acids are regarded as words) into the latent vector space where the geometric relationship can be used to characterize the semantic relationship between the words. And based on the present situation of identifying DTIs by the way of investigating a series of recently published articles (Keiser et al., 2007; Ezzat et al., 2016; Zhang et al., 2016) as well as some review papers (Rognan, 2007; Kombo et al., 2013; Öztürk et al., 2016), we have proposed a novel feature-based computational model for predicting drug-target interactions to enhance prediction performance. The novelty of this proposed work 1) Compared with the end-to-end predictor, we treat DTIs task more flexibly. The protein sequences are regarded as natural language and vectorized by the state-of-art ProtBert model, and drug molecular is transformed by DWT, which is commonly used in signal processing. 2) Calculating the hybrid loss function (contrastive loss and cross-entropy loss), which can make the samples of the same interaction label closer, and the distance between different labels as far as possible and help the predictor achieve higher accuracy.

## 2 Materials and Methods

### 2.1 Benchmark Dataset

Identifying DTIs can be regarded as a supervised prediction task to predict whether a pair of counterparts interact with each other or not in the drug-target networks. In this study, the benchmark dataset was taken from (He et al., 2010). There are mainly two reasons, 1) The information about the DTIs was collected from the DrugBanks, BRENDA, SuperTarget, and KEGG BRITE databases, which included four main drug target proteins of G Protein-coupled receptors (GPCR), enzymes (Ezy), ion channels (Chl), and nuclear receptors (NR). 2) In recent years, many researchers have been proposed to predict DTIs, which are based on this benchmark dataset, and hence will facilitate the comparison under the same condition. It can be summarized as follows:

$$\{\begin{array}{c} S=S_{GPCR-Drug}+S_{Chl-Drug}+S_{Ezy-Drug}+S_{NR-Drug}\\ \begin{array}{c} S_{GPCR-Drug}=S_{GPCR-Drug}^{+}\left(630\right)+S_{GPCR-Drug}^{-}\left(1240\right)\\ S_{Chl-Drug}=S_{Chl-Drug}^{+}\left(1372\right)+S_{Chl-Drug}^{-}\left(2744\right)\;\;\;\;\;\;\;\\ \begin{array}{c} S_{Ezy-Drug}=S_{Ezy-Drug}^{+}\left(2719\right)+S_{Ezy-Drug}^{-}\left(5438\right)\;\;\;\;\\ S_{NR-Drug}=S_{NR-Drug}^{+}\left(82\right)+S_{NR-Drug}^{-}\left(164\right)\;\;\;\;\;\;\;\;\;\;\;\;\;\;\;\; \end{array} \end{array} \end{array}$$ (1)

There are 4,803 drug-target pairs in positive subsets, 2,719 for enzymes, 1,372 for ion channels, 630 for GPCRs, and 82 for nuclear receptors. Negative samples are randomly synthesized by separating each target and drug in S^+^, and none of them appear in the corresponding positive dataset. The proportion of positive samples and negative samples was set as 1:2. For comparison with previously published papers, both our positive and negative samples are consistent with He et al. (He et al., 2010)

Check390 is a dataset constructed by Hu et al. It contains 130 pairs of positive samples from the KEGG database, and 260 negative samples generated using the above method (Hu et al., 2016). Each pair in Check390 cannot be found in S .

### 2.2 Framework of the Constructed Model

**FIGURE 1:**
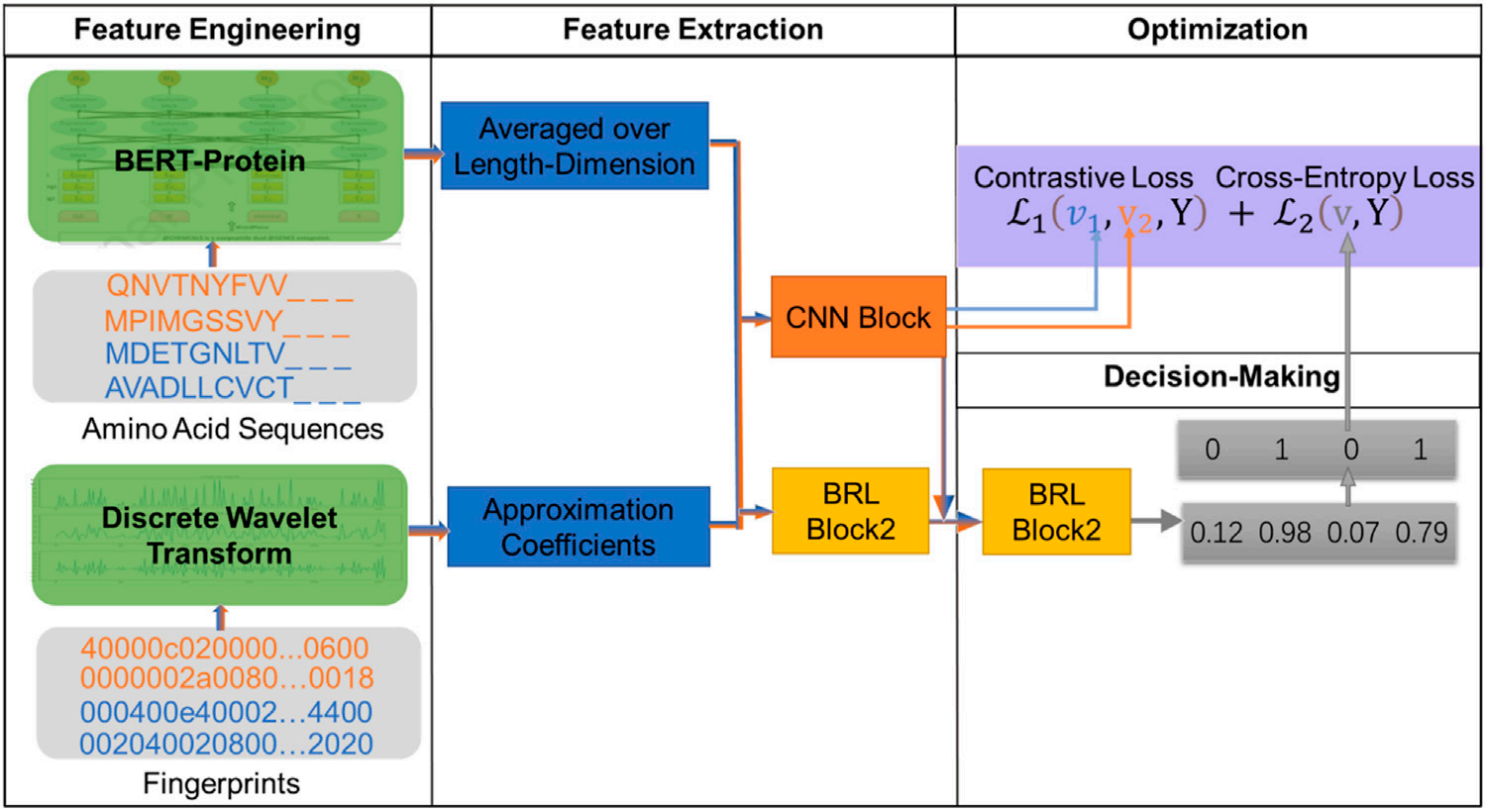
Flowchart of the DTI-BERT model.

In this article, we construct a novel model for DTIs based on large-scale pre-trained Bidirectional Encoder Representations from Transformers (BERT) and the fully connected neural network-based module called the BRL block. Figure 1 showers an overview of the DTIs model. The model has four modules: feature engineering, feature extraction, optimization, and decision-making. Firstly, in the feature engineering module, we use the auto-encoder ProtBert model, which is pre-trained on data from UniRef100 containing 216M protein sequences, to generate embedding vectors for protein sequences. As a result, the proteins can be represented via 1024-D vectors (dimensionality of the features extracted by the ProtBert model). Drug molecular fingerprints are represented by 128-D vectors through semi decomposition process discrete wavelet transform (DWT). Secondly, the 1152-D vectors (a concatenation of protein sequence feature and drug feature) are fed into the feature extraction model to generate interaction information through the first BRL block and CNN Afterwarderwards, in the decision-making module, the second BRL block is used to map interaction features into a unified vector space. The optimization module contains a contrastive loss and a cross-entropy loss. The contrastive loss is used to calculate the interaction information (generated by CNN block), which can reduce the distance between samples with the same label, and increase the distance between samples with different labels, while the cross-entropy loss is computed as the loss of second BRL block, bathes are used to adapt weights in the module during the learning process by minimizing the total loss. At the end of model, we can obtain the interaction score (generated by a softmax layer after second BRL block, and range from 0-1), the pair is interaction if the prediction score is  >0.5 .

#### 2.2.1 Feature Extraction From Protein

Recently, many word-embedding methods have been used for protein feature extraction, for example, Zheng et al. identified the ion channel-drug interaction using both word2vec and node2vec as molecular representation learning methods (Zheng et al., 2021). However, there are still imperfect, like in these word-embedding methods may map every word with their unique vector, therefore this representation is context-independent. With the exponential growth of textual data, major progress has been made in the pre-training language representations (Peng et al., 2019; Bianchi et al., 2021). Bidirectional Encoder Representations from Transformers (BERT) was the first fine-tuning-based representation model (Devlin et al., 2018), which can generate different representations for the same word based on context (Devlin et al., 2018; Nozza et al., 2020).

**FIGURE 2:**
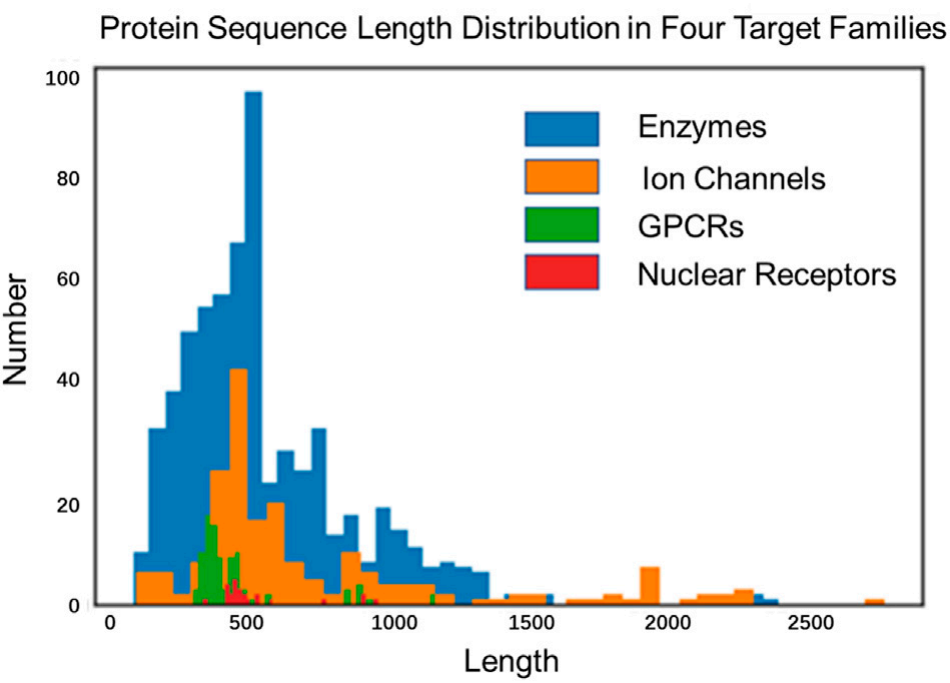
The distribution of protein sequence length.

Almost all sequence-based language models (e.g., context ELMo (Ilić et al., 2018), BERT (Devlin et al., 2018), Xlnet (Yang et al., 2019)) have been promoted the development of processing natural languages successfully, but model architectures and pre-training tasks may not be suitable for representing proteins. The primary reason is that proteins are more variable than sentences in length, and show many interactions in distant positions (due to their 3D structure). The length of English sentences is multiple, usually around 15-30 words (Brandes et al., 2022). Although the length limit of a sentence is not an issue in sentence-level NLP tasks (Dai et al., 2019; Brandes et al., 2022), however, many proteins are more than 20-times longer than nature sentences, reaching an average length of up to 600 residues in drug–the target benchmark dataset and over 20% of the sequences are longer than 1,000. The average length of GPCR, ion channel, enzyme and nuclear receptor are 470, 760, 570 and 540, the distribution of protein sequence length is shown in Figure 2.

For protein sequence representation, Elnaggar et al. released a model called ProtBert, which was trained on UniRef100 datasets (contained 216M protein sequences) (Elnaggar et al., 2021). In the ProtBert model, amino acids are set as single words and protein sequences as sentences. The model can deal with protein sequences up to 40k in length, and can download from: https://github.com/agemagician/ProtTrans (Elnaggar et al., 2021). In the current study, the protein sequence feature can be extracted by ProtBert based on transfer learning (Lee et al., 2019; Noorbakhsh et al., 2020).

The sequence expressed as an amino acid residue may be formulated in the following format:

$$\text{G }=\;R_{1}R_{2}R_{3}\ldots R_{L}$$ (2)

where $R_{1}$ is the first residue in the protein sequence, $R_{2}$ is the second residue, … , $R_{L}$ is the L *- th* residue.

The framework of ProtBert is similar to the original Bert publication, some special encoding symbols like [CLS] and [SEP] remain in the BERT model. [CLS] means classification, is added as the first token in the Bert sequence information. When designing the model, [CLS] token was considered as the representation of subsequent text classification. [SEP] means a separator, for example, the task was sentence-pair regression, the input for BERT consists of the two sentences, that would be separated by a special [SEP] token.

We add a [CLS] token at the beginning of the protein sequence marked as $R_{0}$ , which acts as an aggregate sequence representation and is usually used for sequence classification tasks in the BERT model, and the [SEP] token at the end of the sequence, marked as $R_{L+1}$ .

We get protein features from the last layer of ProtBert, and every amino acid can be converted to a 1024-dimensional vector $\;B_{R_{j}}$ , and the protein can be represented as a feature matrix $P_{BERT}$ :

$$B_{R_{j}}=\;\left[B_{R_{j}}^{1}B_{R_{j}}^{2}\ldots B_{R_{j}}^{\text{i}}\ldots B_{R_{j}}^{1024}\right]$$ (3)

$$P_{BERT}=\left[\begin{array}{ccc} B_{R_{0}}^{1} & \cdots & B_{R_{0}}^{1024}\\ \vdots & \ddots & \vdots \\ B_{R_{L+1}}^{1} & \cdots & B_{R_{L+1}}^{1024} \end{array}\right]$$ (4)

It can be seen from Eqs. 3, 4 that different protein has different size of $P_{BERT}$ . To formulate the protein sequences with the same size mathematics formulation, the matrix was averaged (mean-pooled) over the vertical axis and a 1024-dimensional vector was obtained to be used as a representation of protein named BERT_Mean:

$$\;b_{n}=\frac{\sum_{j=0}^{j=L+1}B_{R_{j}}^{\text{n}}}{L+2}\;\left(1\leq \;n\leq 1024\right)$$ (5)

$$P_{PROT}\;=\;\left[b_{0}b_{2}\ldots b_{n}\ldots b_{1024}\right]$$ (6)

#### 2.2.2 Feature Extraction From Drug Molecule

A drug is saved as an MOL file (a file format that represents a compound in the form of a graph connection table) or SMILES in the database, both formats containing information about the molecule structure, and can be retrieved from the KEGG database (http://www. kegg. jp/kegg/) or ChEMBL (https://www.ebi.ac.uk/chembl/) according to drug IDs. We can also use the MOL file or SMILES as the input of the OpenBabel tool (http://openbabel.org/) to generate the molecular fingerprint file, including FP2, FP3, FP4, and MACSS. FP2 is an enumeration of linear fragments or ring substructures of one to seven connected atoms in a molecule, then maps them to a 256-bit hexadecimal string through a hash function. FP3, FP4, and MACSS use predefined structures to generate fingerprints. FP2 retains more sequence information, we use FP2 as molecular input.

The FP2 molecular fingerprint is represented by a 256-bit hexadecimal string, the hexadecimal char “0∼F” can be converted to the number 0–15, drug molecule is represented as $S_{FP2}$ in the following formulation:

$$S_{FP2}=\;\left[f_{1}f_{2}\ldots f_{256}\right]$$ (7)

In previous studies, the FP2 can be further processed using some transposition functions, and Hu et al. (Hu et al., 2016) and Wang et al. (Wang et al., 2020) have confirmed the effectiveness of applying Discrete Fourier Transform (DFT). DFT can convert molecular fingerprints into frequency-domain values, reflecting the specific characteristics of drug molecules. DFT can freely choose frequency domain or time domain according to the needs of practical applications, however, it cannot obtain information in both cases simultaneously, and we cannot know the time when a signal occurs (in our study, it means sequence position information). To solve the local non-stationary components contained in the FP2, DWT was chosen to extract drug features. Daubechies family is the wavelet basis function in DWT, which can support discrete transformation and have good orthogonality and symmetry compared to other wavelet bases. In this paper, the specified wavelet basis function is used to decompose the fingerprint vector, and the approximation coefficients are used as the wavelet coefficients of the fingerprint vector.

After the transformation of DWT with the Daubechies family, 128 approximation coefficients can be obtained to form a vector:

$$S_{A}=\;\left[a_{1}a_{2}\ldots a_{128}\right]$$ (8)

To better characterize the drug, $S_{A}$ was subjected to a standard conversion as described by the following equation:

$$d_{i}=\;\frac{a_{\text{i}}}{\sum_{j=1}^{128}a_{\text{j}}}$$ (9)

$$D_{DWT}=\;\left[d_{1}d_{2}\ldots d_{128}\right]$$ (10)

And $D_{DWT}$ a 128-dimensional vector is obtained to be used as representation of drug. Finally, through the above several steps, a drug-protein pair can be represented with an 1152-D vector given by:

$$\Phi =\left[\begin{array}{ccc} \Phi_{1} & \Phi_{2} & \begin{array}{ccc} \cdots & \Phi_{i} & \begin{array}{cc} \cdots & \Phi_{1152} \end{array} \end{array} \end{array}\right]$$ (11)

### 2.3 CNN Block

The CNN block includes a convolution layer, a rectified linear unit activation (ReLU), and a max-pooling layer. Instead of using multi-channels, we applied one channel only (Peng et al., 2018). In the convolution layer, apply a convolution kernel with a window size of h*k to extract the DTIs features, then use the rectified linear unit activation function and performed max-pooling to get the most useful interaction feature from the feature matrix subsequently. Through this block, an output of input xis formulated as:

 v=maxpool(f(w⋅x+b)) (12)

where $w\in R^{hk}$ , which is applied to a window of h=18 *,* k=64 to produce a new feature; b∈R is a bias term and f is a non-linear function.

### 2.4 BRL Block

The BRL is built as a special block in the neural network, where data is normalized and then mapped into a specific vector space. This block consists of three layers: a batch-norm layer (BN), a leaky rectified linear activation layer (Leaky ReLU), and a linear layer (Pedregosa et al., 2011).

The input data x is first Batch-normalized, which serves to increase the learning rates further, remove the dropout layer, and apply other modifications afforded by the batch normalization (Ioffe and Szegedy, 2015); then input to the Leaky ReLU activation layer, and finally linearly mapped. BRL block can mathematically be represented as:

X=Linear (LeakyReLU(BN(x)))=W×(LeakyReLU(BN(x)))+B (13)

where x is the input data, the BN transform is applied independently to each dimension of x , W is the weight of the linear layer, and B is the bias of the linear layer. The first BRL block and CNN block are used for capturing both global and local information to represent the drug-protein pair; the second BRL block is used for predicting DTIs.

The BRL block was implemented with PyTorch (version 1.6.0), and a fully connected layer was used for the linear mapping. The parameters of the first BRL block were set as: the number of input neurons and the batch normalized dimensions dimension were both 1,152, and the number of output neurons was set to 128. The parameters of the second BRL block were set as 192 (128-D from the first BRL block and 64-D from the CNN block), and two respectively. A softmax layer is applied after the second BRL block, which is used to generate the prediction score. Other hyperparameters used default values in Pytorch. The source code for the related methods is available on a GitHub repository at: https://github.com/Jane4747/DTI-BERT.

### 2.5 Optimization Module

In this frame, given two vectors $v_{1}$ and $v_{2}$ , input them into the same network in turn, the network will map the inputs to the new vector space where the similarity between two inputs can be evaluated by the distance measure function. Here, Euclidean distance was served as the distance measure, denoted as $D\left(v_{1},\;v_{2}\right)$ :

$$D\left(v_{1},v_{2}\right)=\|v_{1}-v_{22}{\|}_{2}$$ (14)

To make the samples of the same interaction label closer, and the distance between different labels as far as possible, the contrastive loss was applied as the loss function of the CNN network:

$$\;{ℒ}_{1}\left(v_{1},v_{2},Y\right)=\;\frac{1}{2}\left(1-Y\right)D{\left(v_{1},v_{2}\right)}^{2}+\frac{1}{2}Y\left\{max{\left(0,m-D\left(v_{1},v_{2}\right)\right)}^{2}\right\}$$ (15)

where Y=0 if sequences $v_{1}$ and $v_{2}$ , have the same label and Y=1 if they are different, m>0 is a margin. In other words, the margin defines a radius, and dissimilar pairs contribute to the loss function only if their distance is within the radius.

In this study, the second BRL block was used to convert the representation vector v to binary category outputs, the backpropagation algorithm was used to update network parameters, and the cross-entropy loss function was selected as the loss function of the second BRL block:

$${ℒ}_{2}\left(v,Y\right)=\;-Y\;log\left(D\left(v\right)\right)-\left(1-Y\right)log\left(1-D\left(v\right)\right)$$ (16)

Therefore, the loss function of the DTI-BERT model is:

$$ℒ\left(v_{1},v_{2},Y,Y_{1},Y_{2}\right)={ℒ}_{1}\left(v_{1},v_{2},Y\right)+{ℒ}_{2}\left(v_{1},Y_{1}\right)+{ℒ}_{2}\left(v_{2},Y_{2}\right)$$ (17)

where $Y_{1}$ and $Y_{2}$ are the labels of $v_{1}$ and $v_{2}$ .

We implemented our model using Python three and Pytorch (version 1.6.0). Optimizer, training epochs and batch size are set with “Adam”, 70 and 64, respectively. In our work, the optimizing function, “Adam”, use its default parameters value. All codes and trained models can be found via https://github.com/Jane4747/DTI-BERT.

## 3 Results and Discussion

### 3.1 Performance Metrics

The determination of a pair belongs to an interactive drug-target pair or non-interactive drug-target pair, is in the case of single-label classification. The metrics such as accuracy (ACC), sensitivity (Sn), Specificity (Sp), strength (str, the average of Sn and Sp) and Matthew’s correlation coefficient (MCC) are frequently used. The specific formulas are as follows:

$$\left\{ \begin{array}{l} Acc=\frac{TP+TN}{TP+TN+FP+FN}\\ Sn=\;\frac{TP}{TP+FN}\\ Sp=\;\frac{TP}{TP+FP}\\ Str=\;\frac{Sp+Sn}{2}\\ MCC=\;\frac{TP\times TN-FP\times FN}{\sqrt{\left(TP+FP\right)\left(TP+FN\right)+\left(TN+FP\right)\left(TN+FN\right)}} \end{array}\right.$$ (18)

where TP represents the true positive, FN the false negative, TN the true negative, FP the false positive.

### 3.2 Comparison of Several Classic Protein and Drug Feature Extraction Methods

On the protein representation task, auto-encoder models (word2vec and BERT) with different model parameters scales were tested. For the drug representation task, a variety of algorithms in various fields, including natural language processing (word2vec), graph (node2vec and GCN), and signal processing (DWT) were tested.

**FIGURE 3:**
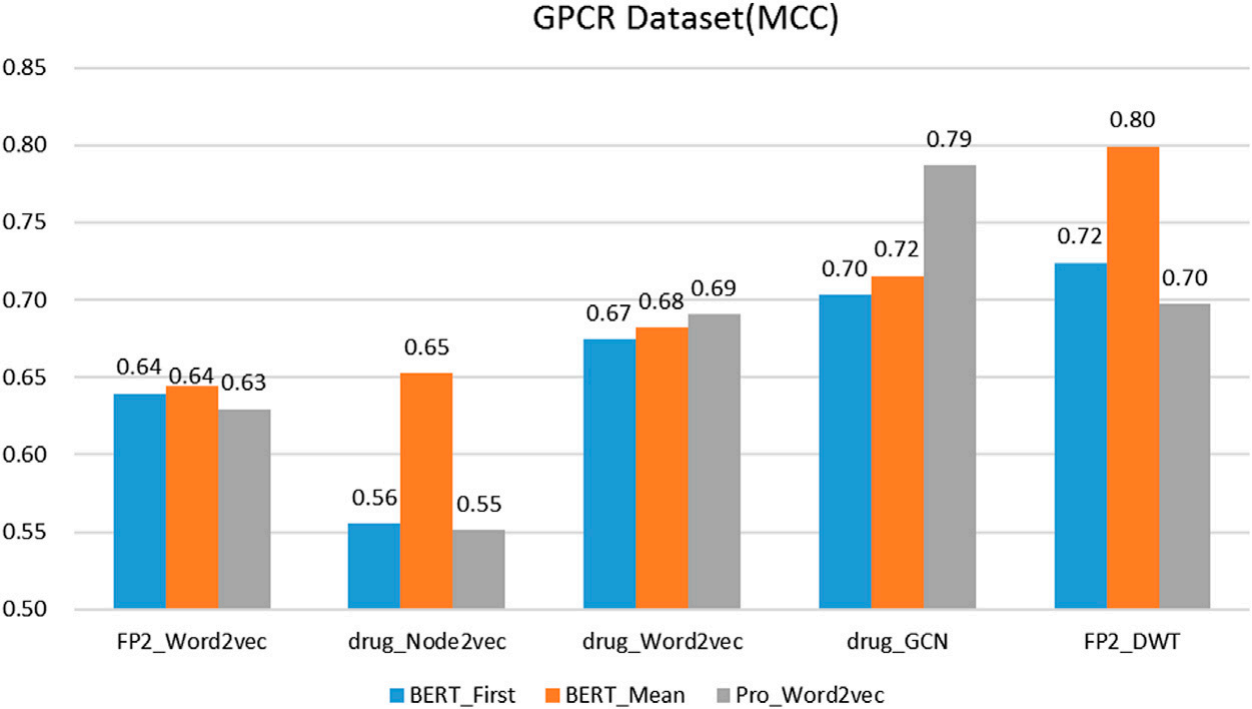
The performance of different protein and drug descriptors on the GPCR dataset.

**FIGURE 4:**
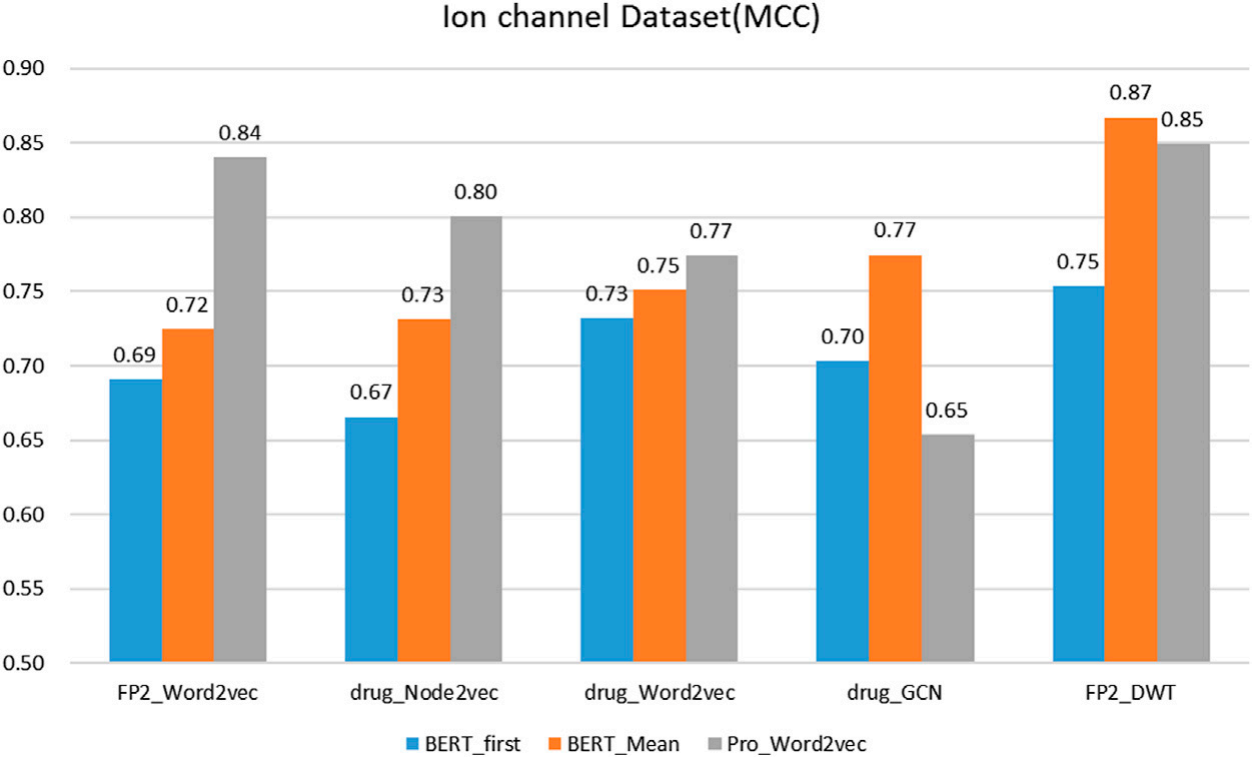
The performance of different protein and drug descriptors on the ion channel dataset.

**FIGURE 5:**
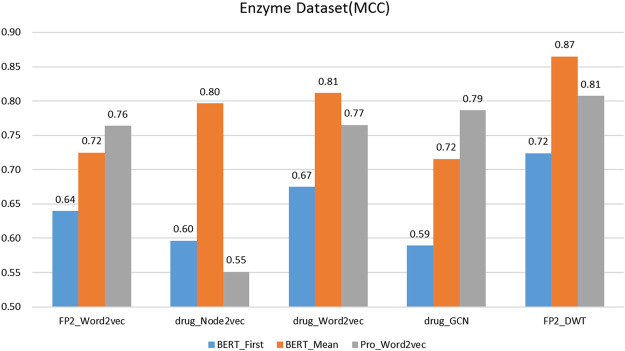
The performance of different protein and drug descriptors on the enzyme dataset.

**FIGURE 6:**
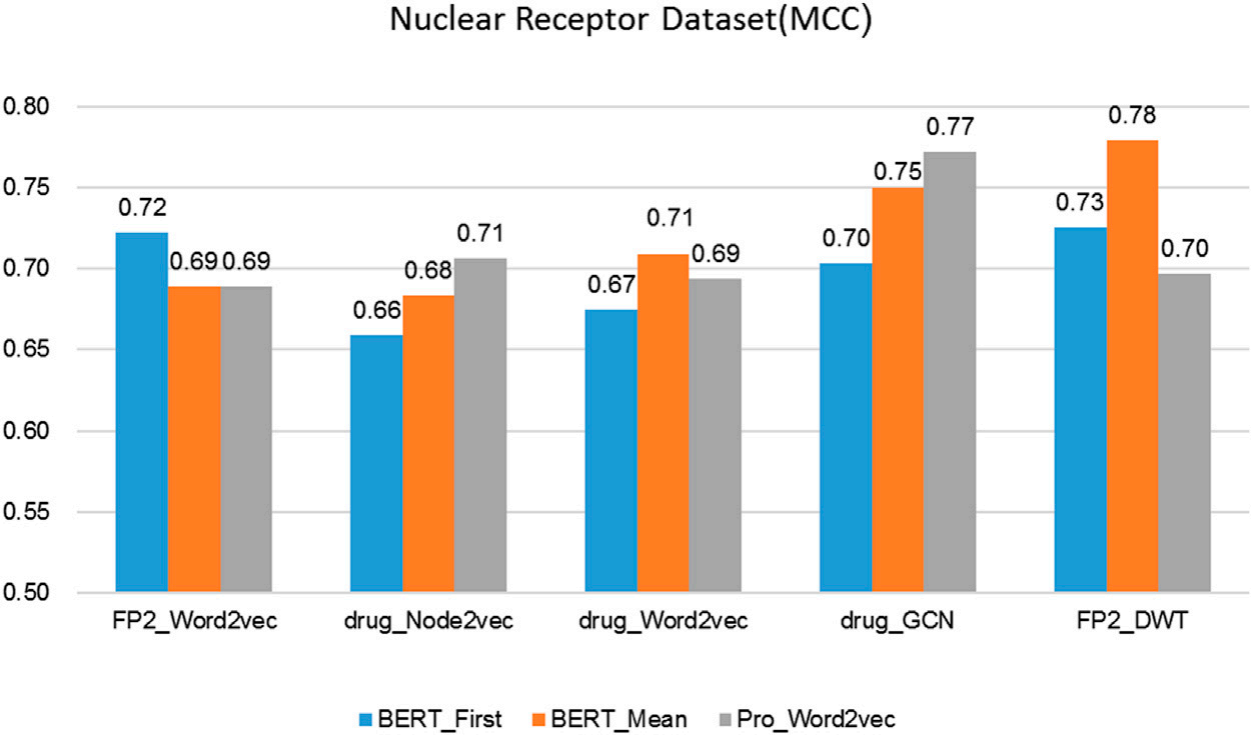
The performance of different protein and drug descriptors on the nuclear receptors dataset.

We evaluated the BERT_Mean + DWT feature extraction method and compared it with several other classic protein and drug feature extraction methods, such as Pr ord2vec (a 64-D vector is obtained to represent the protein, it was extracted by an un-supervised word2vec model and implicated important biophysical and biochemical information (Yang et al., 2018; Zhang et al., 2020), BERT_First (the first row of $P_{BERT}$ is obtained to represented protein, it is a 1024-D vector) (Nambiar et al., 2020), FP2_Word2vec (Jaeger et al., 2018), drug_Node2vec (Grover and Leskovec, 2016; Tetko et al., 2020), drug_Word2vec (Zhang et al., 2020; Zheng et al., 2021), drug_GCN (Chen et al., 2020). Figures 3–6 show the Matthews correlation coefficient (MCC) for the datasets $S_{GPCR-Drug}$ , $S_{Chl-Drug}$ , $S_{Ezy-Drug}$ , and $S_{NR-Drug}$ obtained for each approach in CNN + BRL classifier via 10-fold cross validation.

It was found that BERT_Mean for the proteins and DWT for drugs can improve the performance of the classifier greatly in four datasets. The BERT_Mean + DWT increased capacity for identifying DTIs compared to the using BERT_First, PRO_Word2vec, drug_Node2vec, drug_Word2vec, and drug_GCN, and BERT_Mean can find the most compact and informative features subsets which are deeply hidden in protein sequences. It is showed that word2vec for protein sequences and GCN for drugs in DTIs tasks, could also obtain good prediction results on three datasets ( $S_{GPCR-Drug}$ , $S_{Ezy-Drug}$ , and $S_{NR-Drug}$ ), which inspires us that different protein representation methods need to consider different drug molecule representation methods, which need to be determined experimentally.

### 3.3 Comparison With Some Machine Learning Methods

In order to test the performance of the BRL + CNN and compare it with the existing machine learning methods, we use the same benchmark dataset (listed in Eq. 1) and the same BERT_Mean + DWT feature as the input of the prediction model. The proposed BRL + CNN predictor and other commonly used classifiers provided by the Scikit-learn library, like Multi-Layer Perceptron (MLP) with two hidden layers (Pedregosa et al., 2011) and gradient boosting tree-based ensemble method called LightGBM (LGB) (Ke et al., 2017), were tested via 10-fold cross-validation, the results are listed in Table 1. It was found that the proposed BRL + CNN predictor in this article has better performance than other classifiers in all metrics.

**TABLE 1:** Results of comparison with several traditional machine learning methods on four datasets.

Dataset	Method	Sn(%)	Sp(%)	ACC(%)	Str (%)	MCC
GPCR	MLP	86.8	75.3	82.8	81.5	0.61
GPCR	LightGBM	87.5	80.5	86.3	84.0	0.67
GPCR	BRL + CNN	**89.3**	**91.0**	**90.1**	**90.2**	**0.80**
Ion channel	MLP	93.3	83.1	89.6	88.2	0.77
Ion channel	LightGBM	92.7	89.3	91.7	91.0	0.81
Ion channel	BRL + CNN	**95.9**	**91.4**	**94.7**	**93.7**	**0.87**
Enzyme	MLP	92.2	86.0	90.1	89.1	0.79
Enzyme	LightGBM	92.8	90.5	92.4	91.7	0.83
Enzyme	BRL + CNN	**95.9**	**92.0**	**94.9**	**94.0**	**0.88**
NR	MLP	84.2	76.9	79.9	80.6	0.60
NR	LightGBM	84.4	83.1	82.7	83.8	0.65
NR	BRL + CNN	**92.5**	**85.2**	**89.0**	**88.9**	**0.78**

The best results for each metric are in bold.

### 3.4 Comparison With Existing Predictor

To further demonstrate the power of the DTI-BERT predictor, we compared it with some existing methods. There are some new models for identifying DTIs trained with the datasets established by He et al. (He et al., 2010). For example, Hu et al. proposed a deep learning-based method to predict DTIs by using the information of drug structures and proteins sequences (Hu et al., 2019), this CnnDIT predictor has better prediction performance in predicting DTIs, and it has its own web server. Zhang et al. proposed a random projection ensemble approach DrugRPE to predict DTIs (Zhang et al., 2017), and several random projections build an ensemble REPTress system. In general, the method of fusing multiple predictors outperforms a single predictor. To facilitate comparison, the scores of accuracies (defined in Eq. (18)) obtained by these three predictors (He et al., 2010; Hu et al., 2016; Zhang et al., 2017) based on the benchmark datasets used in He et al. (He et al., 2010) via the 10-fold cross-validation test were listed in Table 2. Comprehensively, the comparative results showed that our model is more accurate than other existing methods.

**TABLE 2:** Performance comparison on four datasets inaccuracy rate.

Method	GPCRs	Ion-Channels	Enzymes	NR
He et al. (2010)	78.5	80.8	85.5	88.4
DrugRPE Zhang et al. (2017)	85.2	89.0	90.0	**91.1**
Hu et al. (2019)	88.4	91.9	94.3	85.7
Our method	**90.1**	**94.7**	**94.9**	89.0

The best results for each metric are in bold.

GPCRs have proved to be one of the most important target families of modern drugs. Identifying the GPR-drug interaction is an important issue in bioinformatics, and a number of researchers have proposed effective predicted methods to identify GPCR-drupredictedions. Our method was also compared with the performance of different methods which predicting GPCR-drug interaction on the training dataset $S_{GPCR-Drug}\;$ over leave-one-out cross-validation, and validated in independent test dataset check390 (Xiao et al., 2013; Hu et al., 2016; Wang et al., 2020; Qiu et al., 2021). The results of the different methods tested on $S_{GPCR-Drug}$ over leave-one-out cross-validation were shown in Table 3. The results of the other eight methods were reported in (Qiu et al., 2021). From Table 3, we can find that the MCC values of our method were 10% higher than others.

**TABLE 3:** Performance comparison on GPCR dataset over leave-one-out cross-validation.

Method	Sn(%)	Sp(%)	ACC(%)	Str (%)	MCC
IGPCR-Drug Xiao et al. (2013)	78.3	91.4	86.9	84.9	0.71
OET-KNN Hu et al. (2016)	77.8	88.7	85.0	83.3	0.67
QuickRBF Hu et al. (2016)	74.8	92.4	86.4	83.6	0.69
SVM Hu et al. (2016)	74.2	92.7	86.4	83.6	0.69
RF Hu et al. (2016)	76.5	92.9	87.3	84.7	0.71
RF + PP Hu et al. (2016)	79.7	92.8	88.3	86.3	0.73
DWKNN(Ensemble) Wang et al. (2020)	81.1	87.1	85.1	84.1	0.67
BOW-GBDT Qiu et al. (2021)	79.8	**93.1**	88.5	86.3	0.74
Our method	**92.2**	92.0	**91.9**	**90.1**	**0.84**

The best results for each metric are in bold.

The generalization ability of machine learning models is usually evaluated through an independent test. The D92M is the GPCR-drug interaction dataset in (Wang et al., 2020), which is applied as a training dataset, and check390 as a validation dataset. The results of the validation test on check390 were listed in Table 4, which demonstrated that our method almost outperform the others across the five metrics, except for BOW-GBDT achieves the highest value of Sp (93.1%). Compared with other state-of-the-art methods, the ACC value of our method is 3.4% higher, the MCC value is 6% higher than the second one. All these results demonstrate the effectiveness of the proposed methods.

**TABLE 4:** Performance comparison on Check390.

Method	Sn(%)	Sp(%)	ACC(%)	Str (%)	MCC
IGPCR-Drug Xiao et al. (2013)	80.8	66.9	71.6	73.9	0.45
OET-KNN Hu et al. (2016)	67.7	84.2	78.7	76.9	0.52
QuickRBF Hu et al. (2016)	76.2	77.7	77.2	77.6	0.52
SVM Hu et al. (2016)	76.2	78.9	78.0	77.6	0.53
RF Hu et al. (2016)	78.5	78.1	78.2	78.3	0.54
RF + PPP Hu et al. (2016)	83.1	79.6	80.8	81.3	0.60
DWKNN Wang et al. (2020)	83.9	80.0	81.3	81.9	0.61
DWKNN(Ensemble) Wang et al. (2020)	83.1	82.7	82.8	82.9	0.63
BOW-GBDT Qiu et al. (2021)	80.0	**90.0**	86.7	85.0	0.70
Our method	**87.1**	89.4	**88.4**	**88.3**	**0.76**

The best results for each metric are in bold.

## 4 Conclusion

In this work, we developed a powerful predictor based on the sequences of proteins and FP2 of drugs. We attempted to use pre-trained BERT to present proteins in DTIs and choose a useful representation for drugs via extensive experiments, including several state-of-art drug descriptions like drug_Word2vec, drug_Node2vec, drug_GCN, FP2_Word2vec, FP2_DWT. The presenting results showed that FP2_DWT is more efficient to present drug molecules than other descriptions. Furthermore, we used the deep learning method to generate interaction information and optimized the predicting network based on contrastive loss and cross-entropy loss, which performed much better than other common machine learning models. Moreover, compared with other existing predictors, DTI-BERT has better prediction performance in different target families of GPCRs, ion channels, enzymes and nuclear receptors, without any help of prior knowledge and handcrafted feature engineering. Overall, DTI-BERT can predict drug-target interactions that achieved high accuracy and we established a prediction web-server for the convenience of the most experienced scientists.

The BERT model has very excellent general capabilities and has very outstanding feature extraction capabilities for DNA sequences (Le et al., 2021) and RNA sequences (Zhang et al., 2021). The DTIs prediction framework proposed in this paper has very good potential for predicting other drug targets as well.

## Data Availability

Publicly available datasets were analyzed in this study. This data can be found here: http://121.36.221.79/dtibert/download.
